# Identification of key genes related to lymphangiogenesis in venous thromboembolism through transcriptomics and verification by RT-qPCR

**DOI:** 10.3389/fmed.2025.1659881

**Published:** 2025-10-24

**Authors:** Yusheng Lin, Jiahan Wu, Pucheng Wang, Xiaodong Lin, Hongwei Yang

**Affiliations:** ^1^Guangxi University of Chinese Medicine, Nanning, China; ^2^Department of Vascular Surgery, Shenzhen Second People's Hospital, Shenzhen, China

**Keywords:** venous thromboembolism, lymphangiogenesis-related genes, immune infiltration, machine learning, key genes

## Abstract

**Background:**

Venous thromboembolism (VTE), a life-threatening cardiovascular disorder, involves complex interactions between thrombosis and immune dysregulation. Lymphangiogenesis-related genes (LRGs) may directly induce thrombosis by regulating endothelial function, the coagulation cascade, or inflammatory signaling pathways. This research was intended to uncover LRG-associated key genes and elucidate their molecular mechanisms in VTE.

**Methods:**

Transcriptomic datasets from public databases were analyzed to identify differentially expressed genes (DEGs) between VTE and control samples. Subsequently, candidate genes were screened by overlapping the DEGs with LRGs obtained from the literature. Functional analysis was then performed on candidate genes. Machine learning algorithms and expression validation were employed to refine key genes. Moreover, gene set enrichment analysis (GSEA), immune infiltration, and regulatory and disease-gene-drug network analyses were performed. Finally, key genes’ expression levels were validated via real-time quantitative polymerase chain reaction (RT-qPCR).

**Results:**

To sum up, 810 DEGs were identified, of which 30 DEGs were selected as candidate genes. Machine learning and expression validation prioritized MYC and NTAN1 as key genes. Functional analysis revealed their enrichment in spliceosome, oxidative phosphorylation, and immune-related pathways. MYC and NTAN1 correlated with regulatory T cells and M2 macrophages. Furthermore, the microRNA (miRNA)-mRNA-transcription factor (TF) network identified MYC as a hub regulated by hsa-miR-449c-5p and JUN. Disease-gene-drug network highlighted cisplatin and olaparib as potential MYC-targeted therapy. RT-qPCR confirmed MYC downregulation and NTAN1 upregulation in VTE (*p* < 0.05), consistent with the bioinformatics results.

**Conclusion:**

This study identified MYC and NTAN1 as pivotal regulators of VTE, bridging thrombotic progression with immune-metabolic dysregulation. The findings provided novel insights into key genes and immunomodulatory therapies for VTE.

## 1 Introduction

Venous thromboembolism (VTE) is the most prevalent type of thrombotic condition, affecting approximately 1 in 12 individuals over 45 (1). With an annual incidence of 300,000 to 600,000 cases in the United States alone, it ranks as the third leading cause of cardiovascular-related mortality (2). VTE usually presents as either deep vein thrombosis (DVT) or pulmonary embolism (PE). Among patients with newly diagnosed VTE, one-third present with concurrent PE, and 25% of PE cases may result in sudden death, underscoring the life-threatening nature of this condition (3, 4).

DVT commonly presents with lower extremity pain, increased skin temperature, swelling, edema, erythema, and tenderness, while PE may cause dyspnea, chest pain, syncope, hemoptysis, hypotension, and tachycardia (5, 6). The clinical manifestations of VTE often lack specificity. Differential diagnoses for DVT include hematoma, cellulitis, congestive heart failure, and superficial thrombophlebitis, whereas PE symptoms may overlap with those of heart failure or myocardial infarction (7). After the initial occurrence of VTE, patients are at risk of recurrence. The core pathological features of VTE, including venous stasis, hypercoagulability, and vascular endothelial injury, jointly promote thrombosis. Although anticoagulant therapy can reduce thrombotic recurrence, these features persist, causing the risk of bleeding to persist. The incidence and recurrence trends in high-risk groups are still worrisome (7). In high-risk groups such as cancer patients, anticoagulants often demonstrate limited efficacy and high recurrence rates, highlighting the urgent need to explore novel mechanisms and therapeutic targets (8, 9).

The pathophysiology of VTE involves complex interactions among endothelial injury, inflammatory responses, and immune dysregulation, though its molecular regulatory networks remain incompletely understood (10, 11). Identifying key driver genes and immune microenvironment characteristics in VTE is critical for achieving early diagnosis and precision treatment.

The lymphatic system is vital for regulating immune cells and plays a significant part in the process of thrombogenesis (12). On one hand, lymphangiogenesis (LYM) promotes tumor metastasis and fosters immunosuppressive microenvironments, correlating with elevated VTE risk in cancer patients (13, 14). On the other hand, lymphangiogenesis-related genes (LRGs) may directly drive thrombosis by modulating endothelial functions (e.g., VE-cadherin expression), coagulation cascades (e.g., fibrinogen activation), or inflammatory signaling (e.g., IL-6/TNF-*α* pathways) (15). However, systematic investigations remain lacking into whether LRGs influence thrombus stability through lymphovascular crosstalk or immune cell infiltration mechanisms (such as neutrophil extracellular traps, NETs) (16). Recent advances in multi-omics technologies (such as transcriptomics and protein–protein interaction networks) offer new insights into LRGs’ molecular functions, though integrated analyses in VTE contexts are absent.

This study integrates transcriptomic data from the GEO database to identify LRG-associated key genes through differential expression analysis and machine learning algorithms. Functional enrichment, immune infiltration profiling, and regulatory network construction are employed to delineate their biological roles. This work establishes for the first time the molecular link between LRGs and immune-metabolic dysregulation in VTE, establishing a theoretical basis for the development of early diagnostic biomarkers and immunomodulatory therapies.

## 2 Materials and methods

### 2.1 The collection of gene data

Gene expression profiles for the training set were obtained from the Gene Expression Omnibus (GEO) database^1^ by downloading the GSE19151 dataset (GPL571). This dataset contained whole blood transcriptomic data from 70 venous thromboembolism (VTE) and 63 control samples. The clinical information of each sample was shown in Supplementary Table 1. The validation set GSE48000 (GPL10558), which was also retrieved from GEO and contained whole blood transcriptomic data, comprised 107 VTE samples and 25 control samples. Additionally, the same analysis was performed in the GSE48000 dataset, and the obtained genes were named DEGs2.

660 lymphangiogenesis-related genes (LRGs) were acquired from the GeneCards database^2^ by searching the keyword “lymphangiogenesis,” based on reference literature (Supplementary Table 2) (17).

### 2.2 Differential expression analysis and candidate gene screening

First, the GSE19151 data was normalized through log_2_ transformation, and a PCA plot was generated. To identify differentially expressed genes (DEGs) between VTE patients and control samples in the training set, the R package “limma” (v 3.56.2) (18) was utilized with criteria of *p* < 0.05 and |log_2_ Fold Change (log_2_FC)| > 0.5. A volcano plot was generated using DEGs in VTE with the use of the R package “ggplot2” (v 3.5.1) (19). The plot labeled the top 10 genes that were up-regulated and the 10 that were down-regulated, ranked by log2FC. Additionally, a heatmap was created using the top 50 up-regulated and top 50 down-regulated genes, with the use of the R package “ComplexHeatmap” (v 2.16.0) (20).

Furthermore, the R package “ggvenn” (v 0.1.9) (21) was utilized to visualize and extract intersection genes between DEGs in VTE and LRGs. The overlapping genes were defined as candidate genes for further functional analyses.

### 2.3 Functional enrichment and protein–protein interaction (PPI) analysis

To explicate the biological functions and signaling pathways linked to the candidate genes, Gene Ontology (GO) and Kyoto Encyclopedia of Genes and Genomes (KEGG) enrichment analyses were carried out with the R package “clusterProfiler”(v 4.8.3) (22), with a significance criterion of p.adjust less than 0.05. The results were subsequently visualized using the R package “enrichplot” (v 1.20.3) (23). Specifically, GO enrichment analysis categorized genes into three functional domains: biological processes (BPs), cellular components (CCs), and molecular functions (MFs), while KEGG analysis identified significant biological pathways. Pathways were ranked based on the count of involved genes, from highest to lowest.

A PPI network was established to investigate interactions at the protein level further. Candidate genes were entered into the Search Tool for the Retrieval of Interacting Genes/Proteins (STRING) database^3^ to predict protein interactions, with a confidence score threshold of > 0.4. The resulting network was visualized using Cytoscape (v 3.10.3) (24), and genes without predicted interactions were excluded.

### 2.4 Identification of key candidate genes with machine learning

Subsequently, to screen candidate genes, three machine learning algorithms were applied.

The Boruta algorithm was an “all-relevant” feature selection method based on random forest. It created a set of “shadow features” (randomly shuffled copies) for the original gene data, then compared whether the importance of each real gene was significantly and stably higher than that of these random shadows. Finally, genes were classified into three categories: “confirmed important,” “rejected,” or “tentative.” Boruta was adopted because it could efficiently screen out all features related to the outcome, not just those genes with the strongest linear relationships. This helped us capture more potential biological signals and avoid missing key genes. Boruta analysis was carried out on the training dataset by utilizing the R package “Boruta” (v 8.0.0) (25). Genes classified as “Confirmed” were designated as Boruta features.

Support vector machine-recursive feature elimination (SVM-RFE) was a “wrapper method” based on the support vector machine (SVM) model. It started by training a model using all genes, then eliminated the least important genes based on weights defined by the model (e.g., coefficient magnitude), retrained the model with the remaining genes, and repeated this recursive loop until only one gene was left. Finally, the importance of genes was ranked based on the order in which they were eliminated. SVM-RFE was chosen because it excelled at handling high-dimensional data (a large number of genes with a small number of samples) and possessed strong nonlinear modeling capabilities. This helped us identify the genes that contributed the most to the model’s classification performance (e.g., distinguishing between VTE and control groups) from complex gene interactions. SVM-RFE was executed on the R package “e1071” (v 1.7.16) (26). The genes corresponding to the model with the highest classification accuracy were selected as SVM-RFE feature genes.

Least absolute shrinkage and selection operator (LASSO) was an “embedded method” for linear regression. During the model training process, it introduced a penalty term (L1 regularization), which automatically shrunk the coefficients of unimportant or redundant features to zero, thereby achieving feature selection. Genes with non-zero coefficients were the ones selected. Lasso was applied because it could not only perform feature selection but also featured regularization to prevent overfitting. This resulted in a simpler and more interpretable linear model, which was well-suited for screening out the core set of genes with the highest predictive value from a large number of candidate genes. The R package “glmnet” (v 4.1.8) (27) was used to perform LASSO regression. The optimal lambda value was determined by minimizing the error through 10-fold cross-validation. Genes selected under the optimal lambda were considered LASSO feature genes.

Finally, the candidate genes intersecting between Boruta, SVM-RFE, and LASSO features were singled out as candidate key genes using the R package “ggvenn” (v 0.1.9). The expression profiles of the candidate key genes were demonstrated in the validation set. The top 3 upregulated and downregulated genes were presented in the validation set.

### 2.5 Expression validation

The Wilcoxon rank-sum test assessed the differential expression levels of candidate key genes between VTE and control samples in the training and validation sets (*p* < 0.05). Box plots were generated to visualize the differential expression levels between VTE and control samples in training and validation sets. Furthermore, genes showing statistically significant and consistent expression trends across both datasets were ultimately identified as key genes.

### 2.6 Gene set enrichment analysis (GSEA) of key genes

Next, GSEA was carried out on the training dataset to investigate the biological functions and pathways associated with the key genes. Spearman correlation analysis was performed between each key gene and all other genes to obtain correlation coefficients. Genes were then ranked in descending order based on these coefficients. Subsequently, GSEA was performed using the R package “clusterProfiler” (v 4.8.3) with the “c2.cp.kegg.symbols.gmt” gene set, which was retrieved from the Molecular Signatures Database (MSigDB)^4^. Enrichment was assessed with thresholds of |Normalized Enrichment Score (NES)| > 1, *q* value < 0.25, and *p* < 0.05. The top 5 pathways ranked by |NES| of each key gene were selected for visualization.

### 2.7 Immune infiltration analysis

To comprehensively evaluate immune infiltration, proportions of 22 immune cell types (28) were calculated for all specimens in the training dataset by using the R package “IOBR” (v 0.99.0) (29) between VTE and control samples. The relative proportions of immune cells were exhibited utilizing the R package “ComplexHeatmap” (v 2.16.0).

Spearman correlation analysis was also conducted via the R package “psych” (v 2.2.9) (30). To explore correlations among immune cell types. Statistically significant correlations were defined as |correlation coefficient (cor)| > 0.3 and *p* < 0.05.

Furthermore, to assess differences in immune cell infiltration between VTE and control groups, the Wilcoxon rank-sum test (*p* < 0.05) was applied. The results were depicted using the R package “ggplot2” (v 3.5.1). Moreover, to further investigate the correlation between key genes and differentially infiltrating immune cells, Spearman correlation analysis was conducted in the training set by utilizing the R package “psych” (v 2.2.9).

### 2.8 Construction of regulatory network

Furthermore, a microRNA (miRNA)-mRNA-transcription factor (TF) network was constructed to investigate the upstream controlling factors of key genes. The miRNAs predicted to target key genes were identified using the miRNA target prediction and functional annotations database (miRDB)^5^ and TargetScan-v9.0^6^ databases. Subsequently, the intersection of miRNAs predicted by both databases was considered the set of key miRNAs for each key gene. TFs regulating the key genes were retrieved from the Comprehensive Database for Regulations of Human Transcription Factors and Their Targets (hTFtarget)^7^ and KnockTF^8^ databases. Afterwards, the resulting TF-mRNA-miRNA network was visualized using Cytoscape (v 3.10.3).

### 2.9 Construction of disease-gene-drug interaction network

Disease and drug interaction analyses were performed to elucidate key genes’ potential pathogenic mechanisms and therapeutic targets. First, the Comparative Toxicogenomics Database (CTD)^9^ was employed to forecast diseases associated with the identified key genes. Disease-gene pairs with an inference score >10 and documented relevance to venous thrombosis were retained for further analysis. Subsequently, the Drug-Gene Interaction database (DGIdb)^10^ was employed to identify potential therapeutic agents targeting the key genes. Finally, a comprehensive disease-gene-drug interaction network was established and visualized using Cytoscape (v 3.10.3).

### 2.10 Real-time quantitative polymerase chain reaction (RT-qPCR) experimental verification

This study collected 5 pairs of whole blood samples from Shenzhen Second People’s Hospital, including 5 control samples (samples 1–5) and 5 VTE samples (samples 6–10). Recruitment for the study took place from June 20, 2025, to June 25, 2025. This study was approved by the Ethics Committee of Shenzhen Second People’s Hospital with the ethics approval number 2025–488-02PJ and conducted in accordance with the ethical principles of the Declaration of Helsinki and the CIOMS International Ethical Guidelines for Health-Related Research Involving Humans. All participants provided written informed consent before sample collection. Additionally, it should be noted that this study did not involve minors. Reverse transcription was performed with the Hifair® III 1st Strand cDNA Synthesis SuperMix. Moreover, RT-qPCR was conducted with the 2 × Universal Blue SYBR Green qPCR Master Mix, with primer sequences detailed in Supplementary Table 3. Meanwhile, GAPDH served as the endogenous control for normalization. Gene expression quantification utilized the 2^-ΔΔCt^ method (31). Graphpad Prism 10 (32) was used for data visualization, with between-group comparisons assessed by two-tailed Student’s t-test. Statistical significance was defined as *p* < 0.05.

### 2.11 Statistical analysis

The R (v 4.2.2) was utilized to conduct statistical analysis. Difference analysis between groups was executed via the Wilcoxon test. We considered a *p*-value lower than 0.05 to be statistically significant. Meanwhile, we provided the purposes and significances of the selection of various computational methods (Supplementary Table 4).

## 3 Results

### 3.1 Identification of 30 candidate genes in VTE

**Figure 1:**
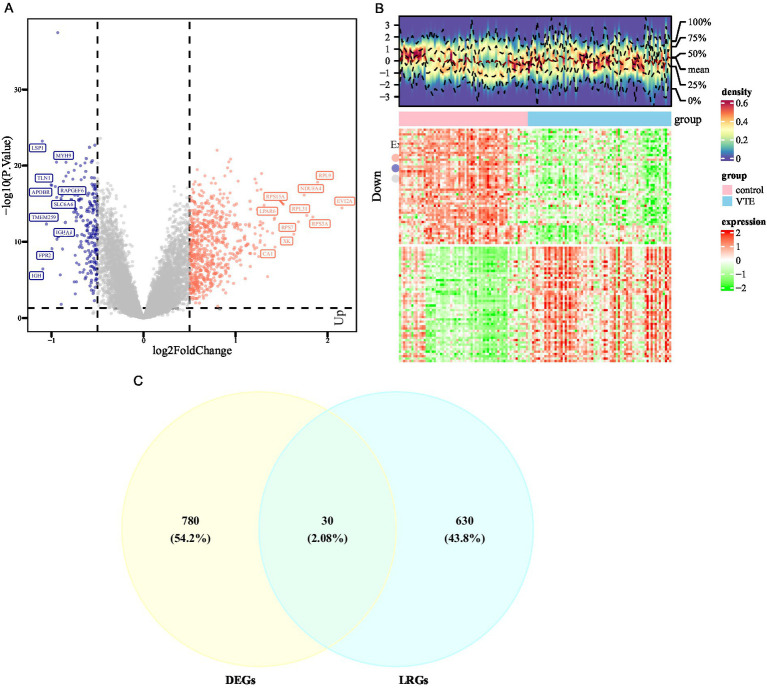
Differential expression analysis of VTE. **(A)** Volcano map of differentially expressed genes between the venous thromboembolism (VTE) group and the normal group, with pink indicating upregulation, blue indicating downregulation, and gray indicating no difference. **(B)** Heat map of differentially expressed genes between the VTE group and the normal group, with red indicating high expression and green indicating low expression. **(C)** The intersection of differentially expressed genes of VTE and pulmonary arterial hypertension (LRGs).

The PCA results showed that the VTE samples and control samples could be separated (Supplementary Figure 1A). Differential expression analysis was conducted between VTE and control in the training set to further investigate the transcriptional alterations in VTE. As a result, 810 DEGs were identified, with 611 genes up-regulated and 199 genes down-regulated in VTE samples (*p* < 0.05, |log_2_FC| > 0.5) (Figure 1A; Supplementary Table 5). Additionally, the heatmap showed the expression patterns of these DEGs between VTE samples and control samples (Figure 1B). In the GSE48000 dataset, 1,992 DEGs were obtained, and 100 genes in DEGs2 overlapped with those in DEGs (Supplementary Figure 1B). Moreover, the intersection analysis of DEGs and LRGs identified 30 candidate genes (Figure 1C). These candidate genes could provide insight into potential therapeutic approaches for VTE.

### 3.2 Functional annotation of candidate genes in VTE

Functional enrichment analyses were performed on 30 candidate genes to decipher the molecular pathways underlying VTE. GO and KEGG analyses exposed their involvement in critical biological processes and disease-related pathways.

**Figure 2:**
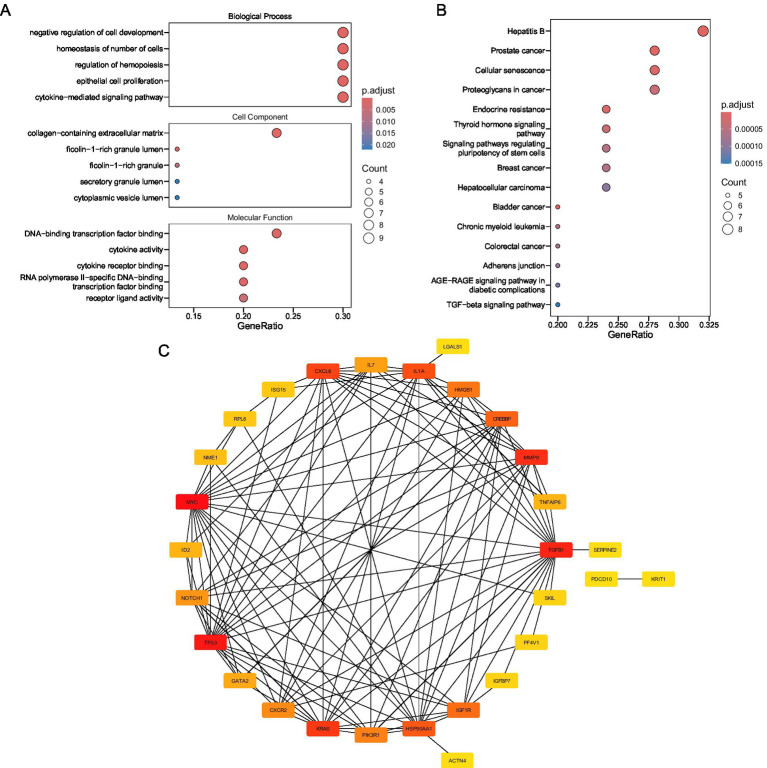
**(A)** Biological function annotation. **(B)** Signal pathway annotation figure. **(C)** Candidate gene protein interaction network diagram.

GO analysis identified 660 significantly enriched terms (p.adjust < 0.05), including 617 BPs, 34 MFs, and 9 CCs (Figure 2A; Supplementary Table 6). Key BP terms were associated with “negative regulation of cell development,” “homeostasis of cell number,” and “cytokine-mediated signaling,” implicating candidate genes in cellular proliferation control and immune regulation. Enriched CC terms highlighted localization to extracellular matrix structures, such as “collagen-containing extracellular matrix” and “secretory granule lumen,” suggesting roles in tissue remodeling and secretory processes. MF terms were dominated by transcriptional and cytokine-related activities, including “DNA-binding transcription factor binding” and “cytokine receptor binding,” indicating regulatory roles in gene expression and immune signaling.

KEGG pathway analysis revealed 94 enriched pathways (p.adjust < 0.05), with prominent associations to oncogenic and metabolic processes (Figure 2B; Supplementary Table 7). Candidate genes were significantly linked to “prostate cancer,” “hepatitis B,” and “cellular senescence,” suggesting shared molecular mechanisms between thrombotic and neoplastic pathologies. Pathways such as “endocrine resistance” and “bladder cancer” further underscore potential roles in therapy resistance and proliferative dysregulation.

A PPI network was also established to explore the functional associations among the 30 candidate genes (Figure 2C). Notably, MYC was found to interact with multiple proteins, including TGFB1, KRAS, and TP53, highlighting its potential central role in the network.

### 3.3 MYC and NTAN1 were identified as key genes through machine learning algorithms and expression validation in VTE

**Figure 3:**
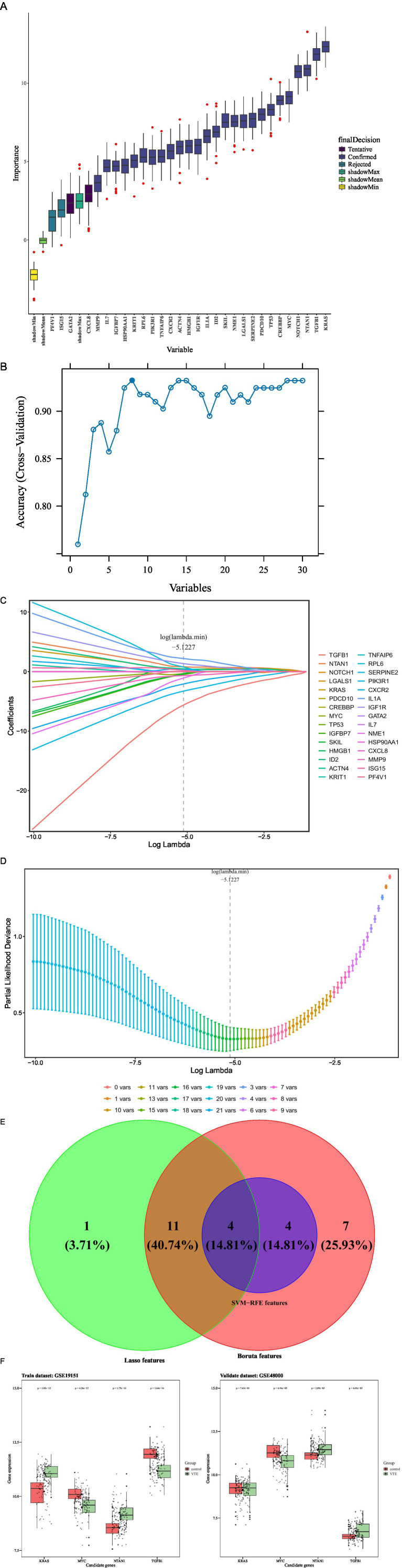
Machine learning. **(A)** Boruta feature importance boxplot. **(B)** SVM-RFE accurate feature relationship diagram. **(C)** Genetic coefficient path diagram. **(D)** Cross-validation error plot. **(E)** Intersection of feature genes predicted by three machine learning algorithms. **(F)** Differential expression levels of candidate key genes.

Machine learning algorithms were applied to refine the candidate genes further to recognize the most relevant genes related to VTE. Specifically, the Boruta algorithm was applied to the training dataset, selecting 26 “Confirmed” genes as Boruta features from the 30 candidate genes (Figure 3A).

Simultaneously, the SVM-RFE algorithm was employed to further identify candidate genes that achieved the best classification performance, identifying eight SVM-RFE features (Figure 3B).

Meanwhile, LASSO regression was applied using the log(lambda.min) value of −5.1227, as determined by 10-fold cross-validation, leading to the selection of 16 LASSO features (Figures 3C,D).

Integrating Boruta, SVM, and LASSO features identified four candidate key genes: TGFB1, MYC, KRAS, and NTAN1 (Figure 3E). In the validation set, TGFB1 and NTAN1 were significantly upregulated in VTE samples, MYC was significantly downregulated, and KRAS showed no significant difference (Supplementary Table 8). Meanwhile, the top 3 upregulated genes in the validation set were IFI27, TMCC2, and GYPB respectively, and the top 3 downregulated genes were ZFP36L2, FOS, and DICER1, respectively, (Supplementary Table 9).

Subsequently, expression validation was implemented on both the training and validation data groups. Among the four genes, MYC and NTAN1 exhibited significantly dysregulated expression in VTE samples compared to controls (*p* < 0.001) and demonstrated consistent trends across datasets (Figure 3F). These findings suggest that MYC and NTAN1 may have important functions in the development of VTE.

### 3.4 Biological pathways associated with MYC and NTAN1 in VTE

GSEA was conducted using the KEGG pathway gene sets to investigate the biological roles of key genes in VTE. MYC and NTAN1 were prioritized for their potential mechanistic contributions to thrombotic processes |NES| > 1, (*q* value < 0.25, and *p* < 0.05).

**Figure 4:**
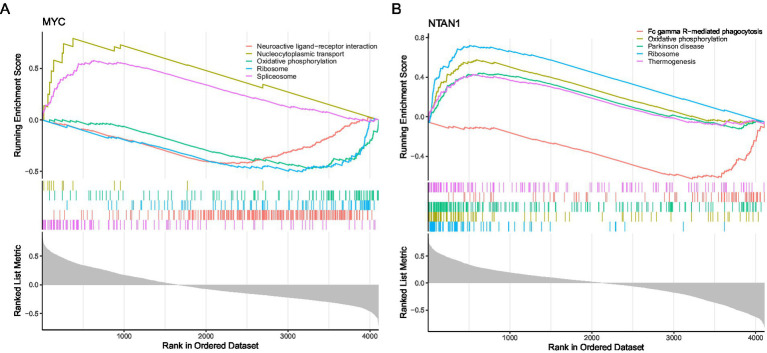
Gene set enrichment analysis. **(A)** Gene set enrichment analysis targeting MYC. **(B)** Gene set enrichment analysis targeting NTAN1.

For MYC, GSEA identified 85 significantly enriched pathways. The top five pathways ranked by |NES| included “spliceosome,” “neuroactive ligand-receptor interaction,” “ribosome,” “oxidative phosphorylation,” and “nucleocytoplasmic transport” (Figure 4A; Supplementary Table 10). The upregulation of “spliceosome” and “nucleocytoplasmic transport” pathways suggested enhanced RNA splicing efficiency and intracellular transport activity in VTE. Conversely, the downregulation of “neuroactive ligand-receptor interactions,” “ribosome biogenesis,” and “oxidative phosphorylation” implied impaired synaptic signaling, protein synthesis, and mitochondrial energy metabolism.

NTAN1-associated GSEA revealed enrichment in 122 pathways, with the top five pathways comprising ribosome, “oxidative phosphorylation,” “Parkinson’s disease,” “Fc gamma R-mediated phagocytosis,” and “thermogenesis” (Figure 4B; Supplementary Table 11). The coordinated upregulation of “ribosome,” “oxidative phosphorylation,” and “thermogenesis” pathways indicated heightened cellular energy production and metabolic activity in VTE. In contrast, suppressing “Fc gamma R-mediated phagocytosis” pointed to compromised immune clearance mechanisms. Notably, the enrichment of Parkinson’s disease-related genes suggested potential overlaps in molecular pathways between neurodegenerative and thrombotic disorders.

### 3.5 Immune infiltration analysis in VTE

**Figure 5:**
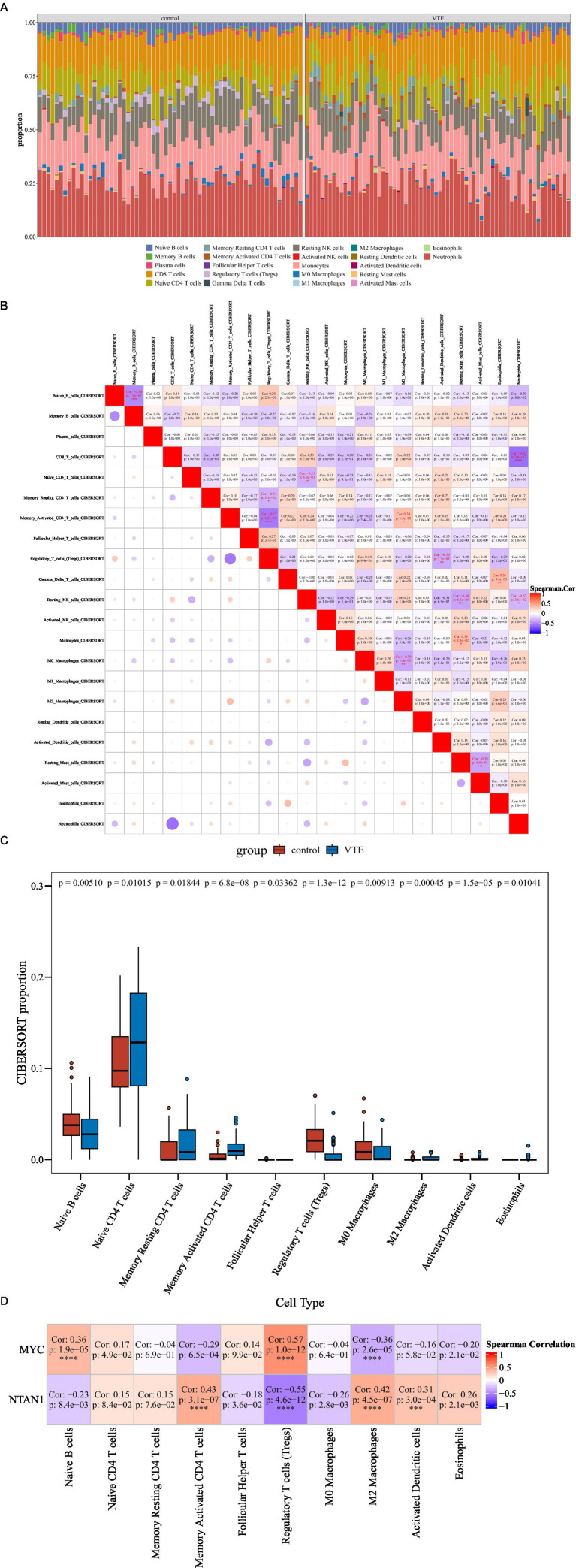
Immune infiltration analysis in VTE. **(A)** Stacked bar chart of CIBERSORT analysis results. **(B)** Heatmap of correlations among 22 immune cell types. **(C)** Differential immune cell boxplot. **(D)** Heatmap of the correlation between differential immune cells and key genes, ***p < 0.001, ****p < 0.0001.

The immune infiltration analysis showed notable differences between VTE samples and control samples. Specifically, the relative proportions of 22 immune cell types were shown in Figure 5A. Spearman correlation analysis of immune cell interactions identified significant associations (|cor| > 0.3, *p* < 0.05) among the 22 immune subsets (Figure 5B). Notably, CD8^+^ T cells displayed a strong negative correlation with neutrophils (cor = −0.62, *p* = 3.6 × 10^−13^).

Comparative analysis revealed 10 differentially infiltrated immune cell types between VTE and control samples (*p* < 0.05), including naive B cells, naive CD4 + T cells, and memory resting CD4^+^ T cells (Figure 5C). Specifically, naive CD4^+^ T cells were elevated in VTE samples, while naive B cells were reduced, suggesting immune dysregulation in VTE.

Moreover, Spearman analysis further uncovered significant associations between key genes (MYC and NTAN1) and differentially infiltrated immune cells (|cor| > 0.3, *p* < 0.05) (Figure 5D). MYC expression positively correlated with regulatory T cells (Tregs) (cor = 0.57, *p* = 1.0 × 10^−12^) and naive B cells (cor = 0.36, *p* = 1.9 × 10^−5^), but negative associations with M2 macrophages (cor = −0.36, *p* = 2.6 × 10^−5^). Meanwhile, NTAN1 showed positive correlations with memory activated CD4^+^ T cells (cor = 0.43, *p* = 3.1 × 10^−7^), M2 macrophages (cor = 0.42, *p* = 4.5 × 10^−7^), and activated dendritic cells (cor = 0.31, *p* = 3.0 × 10^−4^).

These results highlighted the critical interplay between immune dysregulation and thrombotic progression, providing mechanistic insights into VTE pathogenesis and potential immunomodulatory therapeutic targets.

### 3.6 Construction of miRNA-mRNA-TF and disease-gene-drug networks of key genes

**Figure 6:**
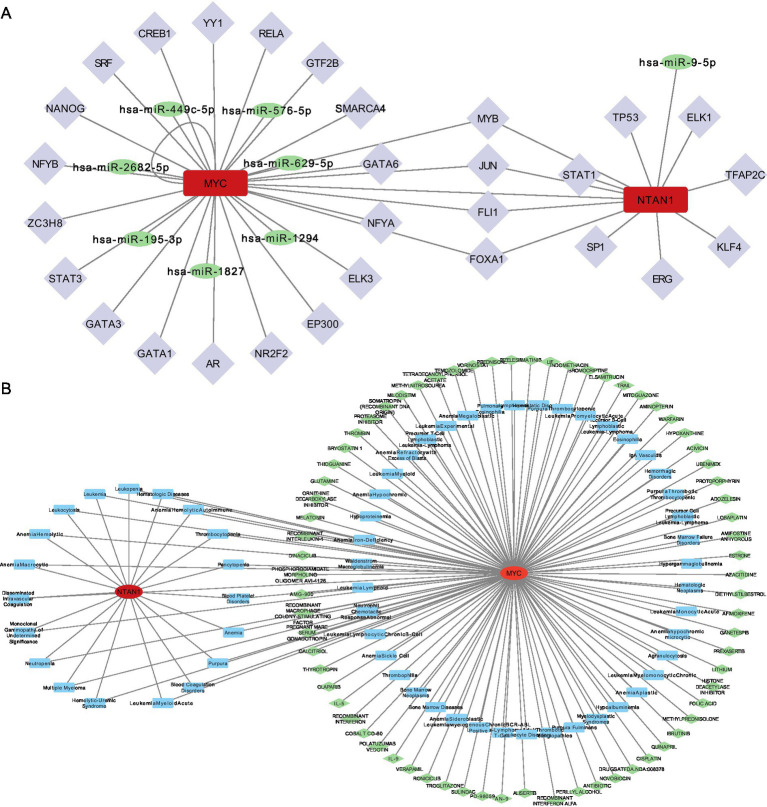
**(A)** TF-mRNA-miRNA interaction relationship network diagram. **(B)** Disease-key gene-drug interaction network.

A miRNA-mRNA-TF network was established to investigate upstream regulatory mechanisms (Figure 6A). Specifically, miRNA predictions for MYC and NTAN1 were obtained from miRDB and TarBase-v9.0 databases, identifying seven and one key miRNAs, respectively. Additionally, TFs for MYC and NTAN1 were retrieved from hTFtarget and KnockTF databases, revealing 23 TFs for MYC and 12 TFs for NTAN1. For example, MYC was targeted by hsa-miR-449c-5p and JUN. Specifically, MYC also exhibited a self-regulatory loop, indicating autoregulation.

A disease–gene–drug network was also constructed and visualized using Cytoscape, with MYC positioned as a central hub connecting thrombotic diseases to candidate therapeutic agents. (Figure 6B).

The CTD identified MYC and NTAN1 as central players in thrombotic disorders, with MYC linked to 62 diseases and NTAN1 associated with 19 diseases. MYC exhibited strong associations with blood coagulation disorders, thrombotic microangiopathies, and thrombophilia, while NTAN1 was enriched in hematologic pathologies such as thrombocytopenia and hemolytic anemia. These findings underscored the dual roles of MYC and NTAN1 in thrombus formation and hematologic dysregulation. Drug-gene interaction analysis revealed MYC as a hub for pharmacologic modulation, with 70 candidate drugs identified (29 approved, 41 not approved). Approved drugs targeting MYC included cisplatin and olaparib, suggesting their therapeutic relevance in thrombotic conditions. In contrast, no drugs were currently predicted to interact with NTAN1, indicating a potential research gap and the need to further explore its druggability.

### 3.7 Expression validation of MYC and NTAN1 expression levels by RT-qPCR

**Figure 7:**
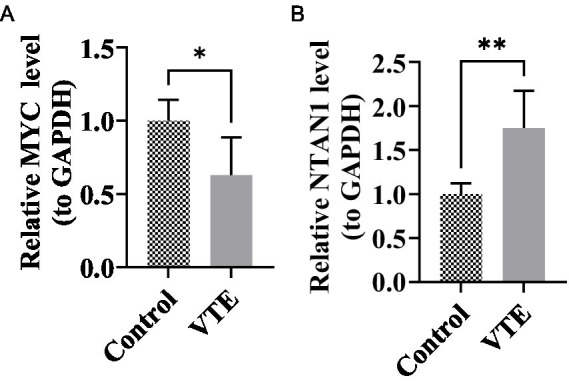
Relative expression levels of MYC and NTAN1 in different groups, *p < 0.05, **p < 0.01. **(A)** MYC **(B)** NTAN1.

To experimentally validate the bioinformatics findings, RT-qPCR was performed to evaluate the expression levels of MYC and NTAN1 in VTE and control samples. Notably, all key genes exhibited significant differential expression (Figures 7A,B) (*p* < 0.05). NTAN1 showed higher expression in VTE samples. In contrast, MYC was down-regulated in VTE samples. These findings confirmed the reliability of the bioinformatics results and reinforced the potential role of MYC and NTAN1 as key genes in VTE.

## 4 Discussion

VTE is a multifactorial disease involving coagulation disorders, endothelial dysfunction, and immune-inflammatory crosstalk. Despite advances in anticoagulant therapy, recurrence rates remain high, necessitating novel biomarkers and therapeutic targets (8, 33). This study, which is among the first of its kind, integrates lymphangiogenesis-related genes (LRGs) with transcriptomic data and employs machine learning algorithms, which may help identify MYC and NTAN1 as potentially key regulatory genes in VTE. Additionally, it suggests possible molecular mechanisms in thrombus formation through immune microenvironment remodeling and dysregulation of metabolic pathways.

MYC (MYC proto-oncogene, bHLH transcription factor) is a critical transcriptional regulator involved in cell proliferation and immune regulation, whose dysregulation may play multifaceted roles in the pathogenesis of VTE (34). Studies have shown that in African clawed frog embryos, MYC maintains the differentiation and migratory capacity of lymphatic endothelial cells (LECs) by regulating the transcription factors Slug/Snail2 and Twist. MYC knockdown leads to hypoplastic embryonic lymphatic networks and generalized edema, whereas exogenous expression of Slug or Twist partially rescues this phenotype. Similarly, in mouse embryos, lymphatic endothelium–specific deletion of MYC reduces lymphatic vessel density by 40% and is accompanied by impaired venous return (35). In pNET cells, MYC overexpression increases VEGFR3 phosphorylation by 1.8-fold and enhances LEC tube formation by 40%, whereas treatment with the MYC inhibitor 10,058-F4 or a VEGF-C neutralizing antibody reduces the lymph node metastasis rate by more than 50% (36). The research, through the integration of gene expression data from individuals with VTE, revealed that MYC could be critical in the development of VTE by modulating cellular growth and metabolic processes. It also indicated that altered MYC expression is closely associated with inflammatory responses and endothelial dysfunction, which may represent one of the key mechanisms underlying VTE development (37). Our study revealed significant downregulation of MYC in peripheral blood samples from VTE patients, which correlated with altered immune cell infiltration patterns and activation of metabolic pathways. This finding contrasts with the well-documented oncogenic role of MYC in malignancies, suggesting the existence of tissue-specific regulatory mechanisms in thrombotic disorders (38, 39).

The observed negative correlation between MYC expression and M2 macrophages, alongside its positive correlation with regulatory T cells (Tregs), suggests that MYC may mediate immune homeostasis disruption in VTE. This aligns with the established role of MYC in tumor immune evasion through Treg activation (40), though its function in thrombosis appears distinct. In this study, MYC expression was negatively correlated with M2 macrophages and positively correlated with regulatory T cells (Tregs). MYC is known to facilitate tumor immune escape by activating Tregs (40). Also, MYC can regulate T - cell proliferation and metabolic reprogramming, which is crucial for T - cell activation and function (41). Additionally, research has found that inhibiting MYC expression in myeloid cells (including macrophages) affects the maturation and pro - tumor activity of tumor - associated macrophages (42). Thus, considering its expression correlations in VTE, it’s speculated that MYC may mediate the disruption of immune homeostasis in VTE. However, as VTE is a thrombosis - related disorder with different pathological processes from tumors, the specific function of MYC in thrombosis may differ from its role in tumor immune escape. GSEA revealed suppressed MYC-associated oxidative phosphorylation pathways, suggesting a potential link between metabolic reprogramming and thrombus formation, a mechanism analogous to cancer-associated thrombosis (43). The inhibition of the oxidative phosphorylation pathway triggers metabolic reprogramming, driving cells such as platelets and macrophages to switch to alternative metabolic pathways like glycolysis (44, 45). This metabolic shift enhances cellular activity and inflammatory responses, thereby promoting thrombus formation.

This study reveals for the first time the aberrant expression pattern of NTAN1 (N-terminal asparagine amidase) in VTE and its underlying molecular mechanisms. Unlike the downregulation trend of MYC in VTE, NTAN1 shows significant upregulation in peripheral blood samples from VTE patients, which may be closely linked to its biological function in post-translational protein modification (46). The N-terminal asparagine amidase encoded by NTAN1, as a key component of the Arg/N-end rule pathway, may influence thrombus formation by regulating the stability of coagulation-related proteins (47). This discovery provides novel insights into understanding the molecular regulatory network of VTE.

From an evolutionary conservation perspective, the highly conserved nature of NTAN1 across 237 species ranging from fruit flies to humans suggests its fundamental role in maintaining coagulation-anticoagulation balance (48). GSEA analysis reveals significant associations between NTAN1 and pathways such as oxidative phosphorylation and Fcγ receptor-mediated phagocytosis, indicating its potential involvement in thrombus clearance through regulating immune cell energy metabolism and phagocytic functions. Notably, the positive correlation between NTAN1 and M2 macrophages may reflect its specific role in modulating anti-inflammatory immune responses, aligning with the characteristic pro-inflammatory/anti-inflammatory imbalance observed in VTE pathological processes (49). M2 macrophages reduce inflammatory responses through the secretion of anti-inflammatory cytokines, including IL-10 and TGF-*β* (50). The findings from this study demonstrated a positive association between NTAN1 and M2 macrophages, leading to the hypothesis that abnormal expression of the NTAN1 gene may alter the anti-inflammatory functions of M2 macrophages by influencing protein degradation and metabolism. This mechanism aligns with the characteristic pro-inflammatory/anti-inflammatory imbalance observed during the pathological progression of VTE (49).

At the clinical translation level, abnormal expression of NTAN1 may serve as a novel biomarker. Its positive correlation with memory-activated CD4 + T cells indicates that this gene may regulate adaptive immunity, thereby affecting the progression of VTE. Although current drug databases have not identified therapeutic agents directly targeting NTAN1, its central role in protein degradation pathways offers a potential therapeutic target for developing novel anticoagulants. Aligned with the individualized treatment principles emphasized in the latest VTE prevention and treatment guidelines (51), NTAN1 expression profiling may provide new molecular evidence for thrombosis risk assessment and precision anticoagulation strategies.

This research identified critical genes associated with the pathological mechanisms of VTE by regulating pathways related to oxidative phosphorylation and ribosome biogenesis. Aberrant activation of the ribosome biogenesis pathway might accelerate thrombus formation by promoting translation efficiency of coagulation factor mRNAs, a process involving precise regulation of ribosomal subunits through nucleocytoplasmic transport mechanisms (52). Dysfunction in the spliceosome pathway could disrupt coagulation homeostasis by generating abnormal transcripts of coagulation-anticoagulation-related genes (e.g., SERPINC1, PROC) through defective RNA splicing. Concurrently, suppressing Fc gamma R-mediated phagocytosis may impair monocyte/macrophage clearance of activated platelets, fostering thrombus progression. Notably, the enrichment of cellular senescence-associated genes (53) reveals a novel mechanistic link between vascular endothelial aging and thrombosis, where the p53-p21 pathway might drive endothelial cells into senescence by regulating ribosome biogenesis checkpoints (54). The significant associations observed in prostate cancer pathways suggest that androgen receptor signaling may influence VTE risk by modulating coagulation factor expression, potentially linked to the gender disparities observed clinically. These pathways exhibit close interactions, such as oxidative stress, which induces cellular senescence and impacts spliceosome function through nucleocytoplasmic transport disturbances (55), forming a complex regulatory network for thrombogenesis.

This study elucidates the critical role of immune cell infiltration in the pathogenesis of VTE. Analyses revealed a significant increase in naive CD4 + T cell proportions in the peripheral blood of VTE patients (*p* < 0.05), accompanied by reduced naive B cell infiltration (56). This immune imbalance may influence thrombus formation by modulating inflammatory responses (57). Neutrophil extracellular trap (NET) formation showed strong association with elevated VTE risk, with released histones and myeloperoxidase directly activating coagulation cascades (58). Studies suggest that CD8 + T cells may participate in thrombus formation and resolution by interacting with cells of the endogenous immune system. In mouse models, selective antibody-mediated depletion of effector memory T cells (TEM), including CD8 + T cells, significantly reduces neutrophil and monocyte recruitment to vascular walls and accelerates thrombus resolution (59). In patients with idiopathic deep vein thrombosis (DVT), CX3CR1-expressing platelet-bound CD8 + lymphocytes are markedly increased and have been proposed as prognostic markers for adverse cardiovascular events (60). While current research indicates that CD8 + T cells may contribute to VTE pathogenesis through multiple mechanisms, their precise role in VTE requires further investigation. This study demonstrates a strong negative correlation between CD8 + T cells and neutrophils, suggesting that CD8 + T cells may suppress neutrophil activation and recruitment, thereby influencing thrombus formation and resolution. However, this hypothesis needs further experimental and clinical validation to establish new theoretical foundations and identify potential therapeutic targets for the prevention and treatment of VTE. Human miRNAs, such as miR-126 and miR-146a, regulate the expression of genes involved in pathways leading to immunothrombosis. Sahu et al. have demonstrated that reduced expression of miR-145 in PBMCs, platelets, vascular endothelial cells, and smooth muscle cells is associated with thrombus development (52). The restoration of normal miR-145 levels in thrombotic animals further reduced thrombosis by decreasing tissue factor levels (52). Therefore, it is crucial to examine shared miRNAs involved in the interplay between inflammation and thrombosis (61). Studies have also shown that serum VEGF levels are downregulated in patients with transient and acute ischemic stroke compared to controls, which correlates with miR-195-5p expression levels. Both miR-195-5p and miR-451a have been shown to target VEGF-A in some experimental settings. This research also indicated that VTE is significantly negatively correlated with miR-195-5p. It is predicted that VEGF-A may be a target gene for miR-195-5p or miR-205-5p.

In breast cancer cells, hsa-miR-195 induces apoptosis by targeting genes such as Bcl-2 and FASN. However, the aberrant activation of MYC can counteract the pro-apoptotic effects of miR-195 by upregulating anti-apoptotic proteins like Bcl-xL. This interaction leads to a decreased sensitivity of tumor cells to chemotherapeutic agents such as doxorubicin, while the overexpression of miR-195 can partially reverse this phenomenon (62). In our study, miR-195 is associated with MYC, and we can infer that miR-195 regulates MYC expression, which may be related to the treatment of venous thromboembolism (63).

In breast cancer cells, hsa-miR-195 induces apoptosis by targeting genes such as Bcl-2 and FASN; however, aberrant activation of MYC can antagonize the pro-apoptotic effect of miR-195 by upregulating anti-apoptotic proteins like Bcl-xL. This interaction reduces tumor cell sensitivity to chemotherapeutic agents such as doxorubicin, while overexpression of miR-195 can partially reverse this phenomenon (62).

In our study, miR-195 was associated with MYC, and we hypothesize that miR-195 regulates MYC expression, which may be relevant to the treatment of venous thromboembolism (63).

In this study, MYC and NTAN1 are identified as key regulatory genes in VTE. Abnormal expression of MYC is closely related to inflammatory responses and endothelial dysfunction, which may be an important link in the pathogenesis of VTE. Our research shows that MYC is significantly downregulated in peripheral blood samples from VTE patients, which is associated with changes in immune cell infiltration patterns and activation of metabolic pathways. Furthermore, MYC expression is negatively correlated with M2 macrophages and positively correlated with regulatory T cells (Tregs), suggesting that MYC may mediate the disruption of immune homeostasis in VTE. NTAN1 is significantly associated with pathways such as oxidative phosphorylation and Fcγ receptor-mediated phagocytosis, indicating that it may participate in thrombus clearance by regulating the energy metabolism and phagocytic function of immune cells. At the clinical translational level, NTAN1 may influence the progression of VTE by regulating adaptive immunity. In our study, miR-195 is associated with MYC, suggesting that miR-195 regulates MYC expression and may be related to the treatment of venous thromboembolism.

This study presents the first evidence connecting LRGs to the immunometabolism of VTE. However, limitations include the small sample size of the retrospective cohort requiring validation of model efficacy through multi-center prospective studies, the lack of animal models and functional experiments to clarify MYC/NTAN1’s causal regulatory relationship via gene knockout, and immune infiltration analysis based on transcriptome deconvolution algorithms that need verification of cell subset specificity through single-cell sequencing. Future research could integrate spatial metabolomics to analyze gene-metabolite interaction networks within the thrombus microenvironment, and explore nanomedicine therapeutic strategies targeting MYC/NTAN1.

The statistical power is severely inadequate, making it difficult to detect true differential expression and biological individual differences (such as age, sex, disease duration, comorbidities, and lifestyle) that cannot be “diluted” in small samples. For instance, if the case group happens to include two “special individuals” (such as those with a very short disease duration or those with other diseases that affect gene expression), their gene expression patterns may deviate from the overall level, directly leading to an exaggeration or masking of the differences between the case and control groups. Furthermore, in the future, consideration should be given to increasing the sample size to enhance the statistical power of the study and the reliability of the conclusions. This can also assist in validating the current findings in larger sample sizes. Due to the use of different R packages, there may be some discrepancies in the results. The choice of threshold can also have a significant impact on the outcomes. The threshold we used is a common one found in most literature, but this does not imply that it is the gold standard.

## 5 Conclusion

This research comprehensively clarified the fundamental molecular mechanisms underlying VTE by integrating bioinformatics and machine learning techniques. Using datasets from the GEO database, we identified 30 candidate genes and further validated MYC and NTAN1 as pivotal genes through Boruta, SVM-RFE, LASSO algorithms, and expression verification. Functional enrichment analysis revealed these genes were significantly associated with cell proliferation regulation, extracellular matrix remodeling, and cancer-related pathways (e.g., prostate cancer, bladder cancer). Gene Set Enrichment Analysis (GSEA) demonstrated that MYC promotes thrombosis via spliceosome and nuclear-cytoplasmic transport pathways, while NTAN1 was linked to dysregulated energy metabolism and impaired immune phagocytosis. Immune infiltration analysis showed significant elevation of regulatory T cells and M2 macrophages in VTE patients, with MYC exhibiting strong correlations to an immunosuppressive microenvironment. Disease-gene-drug interaction network analysis predicted MYC as a potential therapeutic target for antithrombotic drugs. RT-qPCR experiments confirmed the downregulation of MYC and upregulation of NTAN1 in VTE. This study offers new insights into the molecular mechanisms and potential targeted therapies for VTE.

## Data Availability

The datasets [GSE19151, GSE48000] for this study can be found in the [Gene Expression Omnibus (GEO)] [https://www.ncbi.nlm.nih.gov/geo/].

## Footnotes

^1^ https://www.ncbi.nlm.nih.gov/geo/

^2^ https://www.genecards.org/

^3^ http://www.string-db.org/

^4^ https://www.gsea-msigdb.org/gsea/msigdb

^5^ https://mirdb.org/

^6^ https://dianalab.e-ce.uth.gr/tarbasev9

^7^ https://bioinfo.life.hust.edu.cn/hTFtarget#!/

^8^ http://www.licpathway.net/KnockTFv1/

^9^ https://ctdbase.org/

^10^ https://dgidb.org/

## Supplementary material

SUPPLEMENTARY FIGURE 1 **(A)** PCA plot of gene expression data. **(B)** DEGs–DEGs2 overlap.
