# Supplementary material for: Identification of key genes related to lymphangiogenesis in venous thromboembolism through transcriptomics and verification by RT-qPCR
Source: Front Med (Lausanne). 2025 Oct 24;12:1659881. doi: 10.3389/fmed.2025.1659881 (PMC12592171; doi:10.3389/fmed.2025.1659881)
Supplement: Supplementary file 1 [file Data_Sheet_2.ZIP › Supplementary Table/Supplementary Table 3.docx]

Supplementary Table 2 RT-q PCR primer sequences

| **Primers** | **Sequences** | |
| --- | --- | --- |
| MYC F | ACTAACATCCCACGCTCTGA | |
| MYC R | AAACCGCATCCTTGTCCTGT |  |
| NTAN1 F | CACCCGCCTTTGGAGGAA |  |
| NTAN1 R | CCATCCTTTGGGGAGGTCAC |  |
| (Internal control)-GAPDH F | ATGGGCAGCCGTTAGGAAAG |  |
| (Internal control)-GAPDH R | AGGAAAAGCATCACCCGGAG |  |
